# Case Report: The role of BRAF mutation in adjuvant and neoadjuvant treatment of melanoma patients: what is the optimal approach?

**DOI:** 10.3389/fonc.2026.1775350

**Published:** 2026-04-13

**Authors:** Francesca Romana Di Pietro, Rosa Falcone, Sofia Verkhovskaia, Giulia Poti, Cristina Maria Failla, Maria Luigia Carbone, Maria Francesca Morelli, Albina Rita Zappalà, Roberto Morese, Zorika Christiana Di Rocco, Gabriele Piesco, Paolo Chesi, Paolo Marchetti, Federica De Galitiis

**Affiliations:** 1 Oncology and Dermato-oncology Department, Rome, Istituto Dermopatico dell’Immacolata (IDI)-Scientific Institute for Research, Hospitalization and Healthcare (IRCCS), Rome, Italy; 2 Experimental Immunology Laboratory, Istituto Dermopatico dell’Immacolata (IDI)-Scientific Institute for Research, Hospitalization and Healthcare (IRCCS), Rome, Italy; 3 Clinical Trial Center, Istituto Dermopatico dell’Immacolata (IDI)-Scientific Institute for Research, Hospitalization and Healthcare (IRCCS), Rome, Italy; 4 Scientific Direction, Istituto Dermopatico dell’Immacolata (IDI)-Scientific Institute for Research, Hospitalization and Healthcare (IRCCS), Rome, Italy

**Keywords:** adjuvant treatment, BRAF mutation, III stage, melanoma, neoadjuvant strategy

## Abstract

**Background:**

While the benefit of adjuvant therapy is well established in patients with stage IIIB^–^IIID melanoma, its role in stage IIIA disease remains a matter of discussion. This subgroup is generally considered to be at lower risk of recurrence, whereas currently available treatments are associated with potential adverse events, some of which may be severe or irreversible. The presence of a BRAF mutation may assist in identifying patients at increased risk of relapse or those more likely to obtain benefit from adjuvant targeted therapy. Neoadjuvant immunotherapy with checkpoint inhibitors has recently emerged as a promising therapeutic strategy, demonstrating potential advantages over adjuvant treatment. Nevertheless, it may not represent the optimal approach for all patients and should be carefully evaluated on an individual basis, considering specific tumor characteristics such as mutational status to guide treatment selection.

**Case presentation:**

We report the clinical case of a patient with cutaneous melanoma initially diagnosed as stage IIIA, harboring BRAF V600E mutation, who did not receive any adjuvant therapy and developed locoregional recurrence within one year from diagnosis. The patient was subsequently treated with anti-PD-1 neoadjuvant immunotherapy but experienced disease progression at both local and distant sites. Then, targeted therapy was initiated, leading to a rapid clinical and radiological improvement.

**Conclusion:**

Despite significant advances in the treatment of stage III melanoma, both in the adjuvant and, more recently, neoadjuvant settings, some patient subgroups require more tailored approaches. In particular, the presence of BRAF V600 mutation may indicate a more aggressive disease and identify patients who could benefit more from targeted therapy or combined immune checkpoint inhibition than from anti-PD-1 monotherapy.

## Introduction

The introduction of adjuvant therapy in cutaneous melanoma has marked a significant change in the management of stage III disease, reducing the risk of recurrence following complete surgical resection. In recent years, results from phase III clinical trials have led to the approval of adjuvant treatments, either immunotherapy with the anti-PD-1 agents nivolumab or pembrolizumab, or targeted therapy with dabrafenib and trametinib for patients with BRAF V600 mutations. Thesestrategies have significantly improved recurrence-free survival (RFS), offering new therapeutic opportunities but also raising important questions about the management of lower-risk patients and the balance between treatment benefits and potential toxicities (1–3).

Stage IIIA melanoma represents a subgroup with a more favorable prognosis compared to higher stage III subgroups. However, its management remains controversial. While adjuvant treatment with immune checkpoint inhibitors or targeted agents has shown benefits in reducing recurrence risk in stage IIIB-IIIC, the absolute advantage in stage IIIA patients, who are at lower risk of recurrence, is more limited. Consequently, treatment decisions must carefully weigh the potential benefit against the risk of adverse effects (4).

Neoadjuvant therapy in cutaneous melanoma is an emerging treatment approach that involves administering systemic immunotherapy targeting the checkpoint PD-1 molecule prior to surgical resection of the tumor. This strategy has gained increasing interest in resectable stage III melanoma due to its potential to enhance systemic immune responses (5), and clinical trials have demonstrated promising results, with high rates of pathological complete responses (pCR) and improved RFS compared to standard adjuvant therapy with the same anti-PD-1 antibody (6). However, its optimal use, patient selection, and long-term outcomes are still under investigation.

## Case description

We present the case of a 47-year-old woman diagnosed with BRAF V600E-mutated cutaneous melanoma.

In January 2024, she underwent excision of an atypical cutaneous lesion in the right deltoid region, which was found on histological examination to be a non-ulcerated superficial spreading melanoma, tumor-infiltrating lymphocytes absent, with a Breslow thickness of 1.4 mm, classified as pT2a according to the 8th edition of the American Joint Committee on Cancer (AJCC) TNM staging system (7). The patient underwent staging with contrast-enhanced total body computerized tomography (CT), which was negative for disease localization but revealed an enlarged lymph node in the left axillary region that required monitoring. She subsequently underwent breast and axillary lymph nodes ultrasound, which confirmed a 16 mm left lymphadenopathy. Fine-needle aspiration (FNA) was performed on the lymph node, and cytological examination was negative for cancer cells.

In April 2024, the patient underwent wide local excision and biopsy of the right axillary sentinel lymph node, with histological examination revealing a 0.8 mm metastatic deposit within the lymph node. The final staging was pT2pN1a, stage IIIA according to the 8th edition of the AJCC TNM classification.

Mutation status assessment of the primary melanoma was performed, revealing a BRAF V600E mutation and wild-type NRAS.

Postoperative staging revealed a nodular formation in the right breast, for which the patient underwent breast specialist evaluation, mammography, and repeat breast ultrasound, all of which were negative for malignancy.

Considering the time since diagnosis, the stage of the disease, and the thickness of the lymph node metastasis less than 1 mm, the patient was placed under follow-up surveillance only, and no adjuvant treatment was prescribed.

In January 2025, a lymph node ultrasound revealed a 10 mm node with cortical thickening, raising suspicion for disease involvement. The patient was evaluated at our center for the first time and a lymph node biopsy was performed. The histological examination confirmed melanoma recurrence.

**Figure 1:**
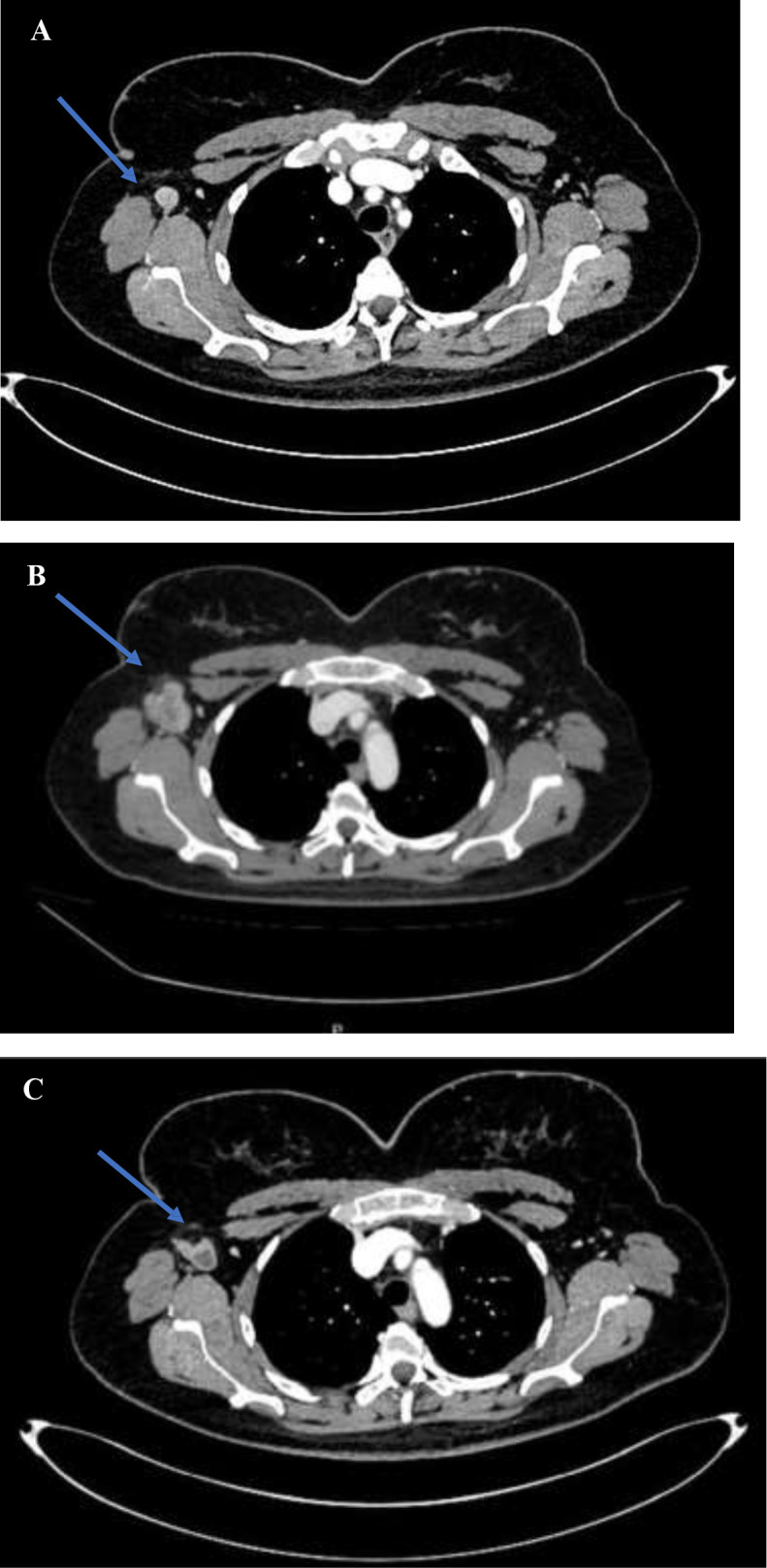
**(A)** CT-Scan performed in February 2025, before neoadjuvant immunotherapy. **(B)** CT scan in May 2025, after three cycles of neoadjuvant immunotherapy. **(C)** CT- Scan performed in August 2025, after three months of target therapy.

At the beginning of 2025, pembrolizumab was available in Italy in the neoadjuvant setting under the Law 648/96, namely an Italian regulation that governs the reimbursement by the Servizio Sanitario Nazionale of innovative medicines that have not yet received full marketing authorization. Instead, the combination of ipilimumab and nivolumab became accessible only later in the year. Therefore, the patient started single-agent immunotherapy after restaging with contrast-enhanced total-body CT (**Figure 1**), which was negative except for the known lymphadenopathy in the right axillary region.

The baseline LDH value before initiation of immunotherapy was normal (190 U/L).

From March to April 2025, the patient received three cycles of neoadjuvant pembrolizumab at a dose of 200 mg every 21 days, experiencing grade 1 diarrhea and nausea as treatment-related toxicities. On May 2025, the patient attended a follow-up visit with a preoperative contrast-enhanced total body CT scan (Figure n.2), which showed the following findings: appearance of a non-calcified nodule in the upper outer quadrant of the right breast measuring 7 mm, another non-calcified nodule in the same quadrant measuring 3 mm, additional bilateral pulmonary nodules, suspicious for progressive disease. Enlargement of the right axillary lymphadenopathy, with the appearance of satellite tissue extending into the subcutaneous region of the axilla was also observed. The lymph node exhibited lobulated contours and necrotic-liquefactive changes, with a size increase from 3x3 cm to 6.6 cm. The satellite tissue measured 23x10 mm. Associated thickening of the fascial planes was consistent with infiltrative changes. Physical examination revealed swelling in the right axillary region.

The LDH values were within in the normal range (196 U/L).

Because of the locoregional and distant disease progression, lymphadenectomy was omitted and assessment of PD-L1 expression was requested to gather all necessary information for determining the next therapeutic steps. Immunohistochemical analysis revealed PD-L1 expression greater than 50%. In Italy, the combination of immunotherapy with ipilimumab plus nivolumab or nivolumab plus relatlimab is available only for melanomas that are negative for PD-L1 expression. An exception is represented by the presence of asymptomatic brain metastases, for which the combination of ipilimumab and nivolumab may be offered. In cases with PD-L1 expression >1%, anti–PD-1 monotherapy is indicated. In our case, given the PD-L1 positivity, but, more importantly, the ongoing progression during immunotherapy as a single agent, a decision was made to initiate first-line targeted therapy. In May 2025, the patient began treatment with encorafenib 450 mg per day plus binimetinib 45 mg twice daily. LDH value before target therapy was normal, i.e. 179 U/L.

After approximately one week, the patient reported significant clinical improvement in the right axillary lymphadenopathy, and at the visit for the second medication dispensation one month later, she showed a complete clinical response in that area. At the follow-up CT scan in August 2025, a radiologic response was observed, with a reduction in size of the lymphadenopathy in the right axilla and at the pulmonary hilum, as well as a significant volumetric reduction of pulmonary nodules (**Figure 1C**). The patient developed grade 1 diarrhea as treatment-related toxicity. Imaging performed in December 2025 showed disease stability.

## Patient perspective

Several effective treatments are now available for stage III melanoma, both in the adjuvant and neoadjuvant settings. However, choosing the most appropriate therapeutic approach is not always evident. In particular, the management of stage IIIA disease and the best treatment option across the different pathological stage III subgroups remain controversial. To guide clinical decisions more effectively, it is important to characterize each case more precisely, without limiting evaluation to stage, but also considering additional factors such as BRAF mutational status.

## Discussion

**Review of literature:** Analysis of BRAF mutational status is a standard procedure in the diagnosis of advanced cutaneous melanoma, and approximately 40–50% of melanoma patients carry an activating mutation in the BRAF gene. BRAF V600 mutations are statistically significantly associated with reduced overall survival (OS) in patients with cutaneous melanoma (8). BRAF V600 mutation leads to constitutive activation of the MAPK/ERK signaling pathway, promoting uncontrolled cell proliferation and tumor growth (9). The most frequent variant, BRAF V600E, accounts for 80% of BRAF-mutated melanomas, followed by V600K and less common subtypes (10). The identification of BRAF mutations has substantial therapeutic implications, as it allows the use ofthe targeted therapy (TT) with BRAF and MEK inhibitors (iBRAF/iMEK), which have significantly improved progression free survival (PFS) and OS in metastatic melanoma patients (11, 12).

Melanomas harboring BRAF mutations are more commonly observed in younger patients, who frequently present with a more aggressive disease course (13).

BRAF V600E-mutant melanomas are characterized by a substantially more immunosuppressive tumor microenvironment (TME) than their BRAF–wild-type counterparts. These tumors display markedly reduced major histocompatibility complex (MHC) expression, leading to impaired presentation of melanoma-associated antigens. They also harbor increased regulatory T-cell (Treg) infiltration in the tumor and elevated concentrations of immunosuppressive cytokines, including vascular endothelial growth factors, interleukin-10, and interleukin-6. Together, these alterations foster a profoundly less immunogenic and more immune-evasive TME (13).

Furthermore, BRAF mutation status, and consequently the use of TT, may modulate the response to immunotherapy (IT), potentially enhancing antitumor efficacy, and combination treatments have been explored (14). Despite translational evidence suggesting that iBRAF/iMEK therapy can potentiate immune responses, their clinical integration with IT has demonstrated only modest benefit.

Despite their aggressive behavior and immunosuppressive tumor microenvironment, BRAF-mutant melanomas still demonstrate meaningful responses to IT and TT (13).

Several studies have investigated the optimal treatment sequence for advanced BRAF-mutant melanoma. In particular, the DREAMseq trial demonstrated a higher objective response rate (ORR) with first-line combination IT compared with IT administered after dabrafenib plus trametinib. In contrast, the ORRs for dabrafenib plus trametinib were similar whether given as first-line or second-line therapy (15). Similarly, the phase II SECOMBIT trial demonstrated that first-line combination IT is superior to first-line TT (16).

Studies have investigated the benefit of either TT or IT with checkpoint inhibitors (ICIs) in the adjuvant setting for patients with a BRAF V600 mutation. To date, the only TT combination approved by the European Medicines Agency and the Italian Medicines Agency for adjuvant use in stage III melanoma is dabrafenib plus trametinib, supported by the results of the COMBI-AD trial (3). As for adjuvant IT, the available options include the anti**-**PD-1 agents nivolumab and pembrolizumab, approved for both stage IIB–IIC and stage III disease (1, 17, 18).

A recent systematic review of real-world data on adjuvant therapy in stage III melanoma was conducted to determine the best option for patients with BRAF V600 mutations. TT showed better RFS and distant metastasis-free survival (DMFS) compared with IT with ICIs, although no apparent difference in OS was observed (19).

Stage IIIA melanoma represents a distinct subgroup within stage III disease, typically characterized by minimal nodal involvement and a lower risk of recurrence compared to more advanced substages. Despite this, the optimal management of these patients remains a topic of debate. While adjuvant therapies with anti-PD-1 agents and BRAF/MEK inhibitors (iBRAF/iMEK) have shown improvements in RFS and DMFS in stage IIIB and IIIC disease, the absolute benefit in stage IIIA patients is limited due to their more favorable baseline prognosis. In a recent analysis by Long et al., adjuvant therapy with anti-PD-1 did not show improved outcomes in patients with stage IIIA melanoma compared to observation. Instead, patients treated with iBRAF/iMEK, irrespective of the size of the lymph node deposit (1 mm or >1 mm), had better RFS and DMFS compared with anti- PD-1 or observation. When nodal metastases were <1 mm, the efficacy of adjuvant therapy was unclear, and the risk–benefit balance remained questionable (20). It should be considered that patients treated with anti-PD-1 IT had worse prognostic clinical features and were numerically more than patients treated with the TT (20).

Neoadjuvant therapy has emerged as an innovative strategy in the management of resectable stage III melanoma, offering a unique opportunity to enhance systemic disease control, prompt the anti-tumor immune response, reduce tumor burden, and obtain early biological insights into treatment efficacy. Studies are available with ICIs and iBRAF/iMEK in BRAF V600 mutated melanoma. ICIs, particularly the combination of nivolumab and ipilimumab, was investigated in different trials. The phase 2 OpACIN-neo trial randomly assigned patients to receive one of three different neoadjuvant dosing regimens and demonstrated high pathological response rates and favorable survival outcomes in the neoadjuvant setting with nivolumab plus ipilimumab, outperforming traditional adjuvant therapy in early-phase trials (21). In the phase 3 NADINA trial, patients with resectable, macroscopic stage III melanoma were randomly assigned to receive either two cycles of neoadjuvant ipilimumab plus nivolumab followed by surgery, or surgery followed by 12 cycles of adjuvant nivolumab. In the neoadjuvant group, only patients who had a partial response or no response received adjuvant therapy, specifically, nivolumab or dabrafenib plus trametinib based on BRAF mutation status. Stratification factors included the presence of a BRAF V600E or V600K mutation (22) Event-free survival (EFS) was significantly longer in the neoadjuvant group than in the adjuvant group; the estimated 12-month EFS rates were 83.7% and 57.2%, respectively. Either patients with BRAF-mutant or with wild-type melanoma had greater benefit when IT was administered before surgery rather than after surgery. Among patients with a BRAF V600E or V600K mutation, the estimated 12-month EFS was 83.5% in the neoadjuvant group and 52.2% in the adjuvant group (22). More recently, neoadjuvant pembrolizumab monotherapy demonstrated clinical and pathological activity, with a favorable toxicity profile and encouraging RFS in the phase II MK-3475 study. The study enrolled patients with melanoma stages IIIB through IVc, and the benefit was demonstrated in both BRAF wild-type and BRAF-mutant melanoma (5).

Both the ipilimumab-nivolumab and pembrolizumab trials used adjuvant anti-PD-1 therapy as the only comparator arm for patients with BRAF-mutated melanoma treated before surgery, without including a comparison with target therapy.

In the PRADO trial, post-neoadjuvant treatment was evaluated and tailored according to the type of pathological response achieved. Patients who achieved a major pathological response (MPR, ≤10% viable tumor) in the largest metastatic lymph node at baseline had therapeutic lymph node dissection (TLND) and adjuvant therapy was omitted. Patients with a partial pathological response (pPR; >10% to ≤50% viable tumor) underwent TLND only, whereas patients with no pathological response (pNR; >50% viable tumor) underwent TLND and adjuvant systemic therapy, either nivolumab or dabrafenib plus trametinib, depending on the presence of a BRAF mutation, or radiotherapy (23).

The role of iBRAF/iMEK has also been evaluated in the neoadjuvant setting in clinical studies. In a phase II trial, patients were randomized to either upfront surgery with consideration for adjuvant therapy (standard-of-care group) or neoadjuvant followed by adjuvant dabrafenib and trametinib. The neoadjuvant regimen consisted of 8 weeks of oral dabrafenib (150 mg twice daily) and oral trametinib (2 mg daily), followed by surgery, and then up to 44 weeks of adjuvant dabrafenib plus trametinib starting 1 week after surgery, for a total treatment duration of 52 weeks. The trial was stopped early after a prespecified interim safety analysis showed that EFS was significantly longer with neoadjuvant plus adjuvant dabrafenib and trametinib compared with the standard-of-care treatment. Moreover, neoadjuvant TT was well tolerated with no occurrence of grade 4 adverse events or treatment-related deaths (24). Similarly, in the Reductor trial, patients with initially unresectable BRAF-mutant melanoma treated with the same neoadjuvant regimen experienced substantial tumor regression, enabling surgical resection in 86% of cases. Complete (R0) resection was achieved in 81% of these patients, with a pCR rate of 29% (25).

Consistent findings were reported in the NeoCombi trial, which confirmed that neoadjuvant dabrafenib plus trametinib in patients with BRAF V600-mutant melanoma yielded high radiologic and pathologic response rates, facilitating surgical resection, and potentially improving long-term prognosis (26). Long-term follow-up data further underscore the potential benefit of this therapeutic approach. At five years, patients with resectable stage III BRAF-mutant melanoma treated with neoadjuvant dabrafenib and trametinib demonstrated RFS of 40%, DMFS of 57%, and OS of 80%. Notably, outcomes were markedly improved among patients who achieved a pCR (27). It is important to note that these studies were limited by relatively small patient cohorts.

The combination of TT with ICIs is also being actively investigated. The phase 2 NeoTrio trial tested whether a target therapy adding dabrafenib/trametinib to neoadjuvant IT with pembrolizumab improved outcomes in resectable stage III BRAF V600-mutant melanoma. Among 60 patients, pathological response rates were 55% with pembrolizumab, 50% with sequential therapy, and 80% with concurrent therapy, but recurrences and toxicity were higher in the target therapy arms. Despite higher initial responses, the combination reduced response quality and safety, indicating that neoadjuvant IT and TT should not be combined for melanoma (28).

In the phase II NEOTIM trial, 59 patients with BRAF-mutant melanoma were randomized to receive either TT alone with vemurafenib plus cobimetinib or triple therapy combining vemurafenib, cobimetinib, and atezolizumab. The study demonstrated that target therapy alone resulted in a higher major pathological response (MPR) rate compared to triple therapy (45% vs. 34%) (13). However, the 12-month RFS favored the triple combination (86% vs. 78%). In the phase II NeoACTIVATE trial, 15 patients with BRAF-mutant melanoma were treated with vemurafenib, cobimetinib, and atezolizumab, achieving an MPR of 66.7% (13). Based on the available studies in the literature, to date only IT with pembrolizumab or the combination of ipilimumab and nivolumab is indicated in the neoadjuvant setting.

**Considerations**: In the adjuvant setting, robust evidence supports the use of both TT and ICIs in patients with BRAF-mutant melanoma, enabling the identification of subgroups that may have greater benefit from one approach over the other. However, several questions remain unresolved, including the optimal management of stage IIIA disease, although BRAF mutation status may serve as a valuable factor in guiding therapeutic decisions.

In contrast, comparable data in the neoadjuvant setting are lacking. Studies investigating TT have included a limited number of patients, and some studies were terminated early, precluding a meaningful comparison with ICIs-based trials. Nonetheless, BRAF-mutant melanomas exhibit distinct biological features and a more aggressive clinical behavior compared with BRAF–wild-type tumors, suggesting that these patients may require a tailored approach when selecting the optimal neoadjuvant strategy.

In the context of our clinical case, the detection of a BRAF mutation likely influenced both the disease course and treatment outcomes. Considering current evidence and clinical experience, the presence of a BRAF mutation represents a key biological determinant in stage III melanoma, with significant implications for therapeutic decision-making and personalized treatment strategies.

## Conclusions

The management of patients with resectable stage III melanoma has benefited from additional therapeutic options, but this has also made the selection of the optimal treatment approach more complex.

Characteristics that can guide clinicians in treatment selection include the risk of recurrence, determined by disease stage and histopathological features such as Breslow thickness and the extent of nodal involvement, and the molecular tumor profile, in particular BRAF mutation status.

The management of stage IIIA melanoma remains a subject of debate, particularly regarding whether patients should receive adjuvant therapy, given their relatively low risk of recurrence and the potential side effects of treatment, especially with anti–PD-1 agents. In this patient group, BRAF mutational status appears to play an important role, as target therapy has demonstrated benefits in BRAF-mutated patients, with adverse events generally being reversible.

Within the context of stage IIIA, another unresolved issue is the management of patients with lymph node metastases <1 mm, who were excluded from the pivotal registration studies.

Regarding the timing of surgery, it is necessary to consider that not all patients respond to neoadjuvant therapy, compromising the feasibility of the next radical surgery. Identification of biomarkers of resistance to therapies is necessary to select those patients who are most likely to benefit from preoperative therapy and neoadjuvant treatment, in respect to the patients who should proceed directly to surgery. Controlled studies comparing neoadjuvant treatments in the BRAF-mutated population are needed to identify the optimal neoadjuvant therapy for these patients.

## Data Availability

The raw data supporting the conclusions of this article will be made available by the authors, without undue reservation.

## References

[1] Eggermont AMM Blank CU Mandala M Long GV Atkinson V Dalle S . Adjuvant pembrolizumab versus placebo in resected stage III melanoma. N Engl J Med. (2018) 378(19):1789–1801. doi: 10.1056/NEJMoa1802357 29658430

[2] Weber J Mandala M Del Vecchio M Gogas HJ Arance AM Cowey CL . Adjuvant nivolumab versus ipilimumab in resected stage III or IV melanoma. N Engl J Med. (2017) 377(19):1824–1835. doi: 10.1056/NEJMoa1709030 28891423

[3] Long GV Hauschild A Santinami M Atkinson V Mandalà M Chiarion-Sileni V . Adjuvant dabrafenib plus trametinib in stage III BRAF-mutated melanoma. N Engl J Med. (2017) 377(19):1813–1823. doi: 10.1056/NEJMoa1708539, PMID: 28891408

[4] Ascierto PA Borgognoni L Botti G Guida M Marchetti P Mocellin S . New paradigm for stage III melanoma: from surgery to adjuvant treatment. J Transl Med. (2019) 17(1):266. doi: 10.1186/s12967-019-2012-2, PMID: 31412885 PMC6693227

[5] Patel SP Othus M Chen Y Wright GP Yost KJ Hyngstrom JR . Neoadjuvant-adjuvant or adjuvant-only pembrolizumab in advanced melanoma. N Engl J Med. (2023) 388(9):813–823. doi: 10.1056/NEJMoa2211437, PMID: 36856617 PMC10410527

[6] Mangla A Lee C Mirsky MM Wang M Rothermel LD Hoehn R . Neoadjuvant dual checkpoint inhibitors vs anti-PD1 therapy in high-risk resectable melanoma: A pooled analysis. JAMA Oncol. (2024) 10(5):612–620. doi: 10.1001/jamaoncol.2023.7333, PMID: 38546551 PMC10979364

[7] Gershenwald JE Scolyer RA Hess KR Sondak VK Long GV Ross MI . (2017). Melanoma staging: Evidence-based changes in the American Joint Committee on Cancer eighth edition cancer staging manual. CA Cancer J Clin 67(6):472–492. doi: 10.3322/caac.21409. PMCID: PMC5978683, PMID: 29028110 PMC5978683

[8] Ottaviano M Giunta EF Tortora M Curvietto M Attademo L Bosso D . BRAF gene and melanoma: back to the future. Int J Mol Sci. (2021) 22(7):3474. doi: 10.3390/ijms22073474, PMID: 33801689 PMC8037827

[9] Dhomen N Marais R . New insight into BRAF mutations in cancer. Curr Opin Genet Dev. (2007) 17:31–9. doi: 10.1016/j.gde.2006.12.005, PMID: 17208430

[10] Nepote A Avallone G Ribero S Cavallo F Roccuzzo G Mastorino L . Current controversies and challenges on BRAF V600K-mutant cutaneous melanoma. J Clin Med. (2022) 11(3):828. doi: 10.3390/jcm11030828, PMID: 35160279 PMC8836712

[11] Robert C Grob JJ Stroyakovskiy D Karaszewska B Hauschild A Levchenko E . Five-year outcomes with dabrafenib plus trametinib in metastatic melanoma. N Engl J Med. (2019) 381(7):626–636. doi: 10.1056/NEJMoa1904059 31166680

[12] Larkin J Ascierto PA Dréno B Atkinson V Liszkay G Maio M . Combined vemurafenib and cobimetinib in BRAF- mutated melanoma. N Engl J Med. (2014) 371(20):1867–76. doi: 10.1056/NEJMoa1408868 25265494

[13] Braden J Conway JW Wilmott JS Scolyer RA Long GV da Silva IP . Do BRAF-targeted therapies have a role in the era of immunotherapy? ESMO Open. (2025) 10:105314. doi: 10.1016/j.esmoop.2025.105314, PMID: 40543211 PMC12221719

[14] Ribas A Lawrence D Atkinson V Agarwal S Miller WH Carlino MS . Combined BRAF and MEK inhibition with PD-1 blockade immunotherapy in BRAF-mutant melanoma. Nat Med. (2019) 25(6):936–940. doi: 10.1038/s41591-019-0476-5 PMC8562134 31171879

[15] Atkins MB Lee SJ Chmielowski B Tarhini AA Cohen GI Truong TG . Combination dabrafenib and trametinib versus combination nivolumab and ipilimumab for patients with advanced BRAF-mutant melanoma: the DREAMseq trial-ECOG-ACRIN EA6134. J Clin Oncol Off J Am Soc Clin Oncol. (2023) 41(2):186–197. doi: 10.1200/JCO.22.01763, PMID: 36166727 PMC9839305

[16] Ascierto PA Casula M Bulgarelli J Pisano M Piccinini C Piccin L . Sequential immunotherapy and targeted therapy for metastatic BRAF V600 mutated melanoma: 4-year survival and biomarkers evaluation from the phase II SECOMBIT trial. Nat Commun. (2024) 15(1):146. doi: 10.1038/s41467-023-44475-6, PMID: 38167503 PMC10761671

[17] Larkin J Del Vecchio M Mandalá M Gogas H Arance Fernandez AM Dalle S . Adjuvant nivolumab versus ipilimumab in resected stage III/IV melanoma: 5-year efficacy and biomarker results from checkMate 238. Clin Cancer Res. (2023) 29(17):3352–61. doi: 10.1158/1078-0432.CCR-22-3145, PMID: 37058595 PMC10472092

[18] Luke JJ Rutkowski P Queirolo P Del Vecchio M Mackiewicz J Chiarion-Sileni V . Pembrolizumab versus placebo as adjuvant therapy in completely resected stage IIB or IIC melanoma (KEYNOTE-716): a randomised, double- blind, phase 3 trial. Lancet. (2022) 399(10336):1718–29. doi: 10.1016/S0140-6736(22)00562-1 35367007

[19] Amaral T Nanz L Higuita LMS Ascierto P Berking C Couselo EM . A comparison of real-world data on adjuvant treatment in patients with stage III BRAF V600 mutated melanoma - Results of systematic literature research. Eur J Cancer. (2025) 215:115160. doi: 10.1016/j.ejca.2024.115160, PMID: 39673834 PMC7618644

[20] Grover P Lo SN Li I Kuijpers AMJ Kreidieh F Williamson A . Efficacy of adjuvant therapy in patients with stage IIIA cutaneous melanoma. Ann Oncol. (2025) 36(7):807–818. doi: 10.1016/j.annonc.2025.03.021 40204154

[21] Rozeman EA Menzies AM van Akkooi ACJ Adhikari C Bierman C van de Wiel BA . Identification of the optimal combination dosing schedule of neoadjuvant ipilimumab plus nivolumab in macroscopic stage III melanoma (OpACIN-neo): a multicentre, phase 2, randomised, controlled trial. Lancet Oncol. (2019) 20(7):948–960. doi: 10.1016/S1470-2045(19)30151-2, PMID: 31160251

[22] Blank CU Lucas MW Scolyer RA van de Wiel BA Menzies AM Lopez-Yurda M . Neoadjuvant Nivolumab and Ipilimumab in Resectable Stage III Melanoma. N Engl J Med. (2024) 391(18):1696–1708. doi: 10.1056/NEJMoa2402604 38828984

[23] Reijers ILM Menzies AM van Akkooi ACJ Versluis JM van den Heuvel NMJ Saw RPM . Personalized response-directed surgery and adjuvant therapy after neoadjuvant ipilimumab and nivolumab in high-risk stage III melanoma: the PRADO trial. Nat Med. (2022) 28(6):1178–1188. doi: 10.1038/s41591-022-01851-x, PMID: 35661157

[24] Amaria RN Prieto PA Tetzlaff MT Reuben A Andrews MC Ross MI . Neoadjuvant plus adjuvant dabrafenib and trametinib versus standard of care in patients with high-risk, surgically resectable melanoma: a single-centre, open-label, randomised, phase 2 trial. Lancet Oncol. (2018) 19(2):181–193. doi: 10.1016/S1470-2045(18)30015-9, PMID: 29361468

[25] Blankenstein SA Rohaan MW Klop WMC van der Hiel B van de Wiel BA Lahaye MJ . Neoadjuvant cytoreductive treatment with BRAF/MEK inhibition of prior unresectable regionally advanced melanoma to allow complete surgical resection, REDUCTOR: A prospective, single-arm, open-label phase II trial. Ann Surg. (2021) 274(2):383–389. doi: 10.1097/SLA.0000000000004893, PMID: 33843797

[26] Long GV Saw RPM Lo S Nieweg OE Shannon KF Gonzalez M . Neoadjuvant dabrafenib combined with trametinib for resectable, stage IIIB-C, BRAFV600 mutation-positive melanoma (NeoCombi): a single- arm, open-label, single-centre, phase 2 trial. Lancet Oncol. (2019) 20(7):961–971. doi: 10.1016/S1470-2045(19)30331-6, PMID: 31171444

[27] Menzies AM Lo SN Saw RPM Gonzalez M Ch'ng S Nieweg OE . Five-year analysis of neoadjuvant dabrafenib and trametinib for stage III melanoma. Ann Oncol. (2024) 35(8):739–746. doi: 10.1016/j.annonc.2024.05.002, PMID: 38754780

[28] Long GV Carlino MS Au-Yeung G Spillane AJ Shannon KF Gyorki DE . Neoadjuvant pembrolizumab, dabrafenib and trametinib in BRAFV600-mutant resectable melanoma: the randomized phase 2 NeoTrio trial. Nat Med. (2024) 30(9):2540–2548. doi: 10.1038/s41591-024-03077-5, PMID: 38907159 PMC11405264

